# Red blood cell transfusion is associated with an increased mortality in critically ill surgical patients

**DOI:** 10.1186/cc14414

**Published:** 2015-03-16

**Authors:** A Piriyapatsom, O Chaiwat, S Kongsayreepong

**Affiliations:** 1 Siriraj Hospital, Bangkok, Thailand

## Introduction

The aim of this study is to explore the association between red blood cell transfusion (RBCT) and mortality in Thai critically ill surgical patients.

## Methods

This study was a part of the multicenter, prospective, observational study performed in nine surgical intensive care units (SICUs) across the nation between April 2011 and November 2012 [1]. This study included adult patients admitted to the SICUs after surgery. Patients were categorized into transfusion and no transfusion groups according to whether or not they received RBCT at any time during SICU stay. Demographic data, clinical outcomes as well as SICU and hospital length of stay (LOS) and SICU and hospital mortality were collected. Patients were followed for up to 28 days or until discharge from the SICUs. The primary endpoint was hospital mortality. Data were compared between groups and logistic regression analysis was performed to determine whether RBCT was an independent risk factor of hospital mortality. In addition, patients were matched between groups based on the propensity score of the requirement of RBCT and were then compared.

## Results

Overall, 968 of 2,374 (40.8%) patients received RBCT. Transfused patients, when compared with those without RBCT, had more frequency of admission after emergency surgery, higher APACHE II score, higher SOFA score, higher number of organ dysfunctions and lower hemoglobin level at admission. When compared with patients without RBCT, those with RBCT had more frequency of all adverse events including infection, AKI, ALI/ARDS and MI, and longer SICU and hospital LOS. Both SICU and hospital mortality were also higher in the transfusion group compared with the no transfusion group (9.4% vs. 1.6% and 13.7% vs. 3.6%, both *P *< 0.001, respectively). The logistic regression analysis showed that RBCT was an independent risk factor of hospital mortality with odds ratio of 1.60 (95% CI 1.05 to 2.45). In the propensity-score matched cohort of 852 patients, when compared with patients without RBCT, transfused patients had more frequency of adverse events including infection and AKI, longer SICU and hospital LOS and higher hospital mortality (7.5% vs. 4.0%, *P *= 0.027).

## Conclusion

This study showed that RBCT was associated with increased morbidity and mortality in critically ill surgical patients. These results supported the restrictive strategy of RBCT suggested by more recent studies.
